# Migration Drives the Replacement of *Xanthomonas perforans* Races in the Absence of Widely Deployed Resistance

**DOI:** 10.3389/fmicb.2022.826386

**Published:** 2022-03-18

**Authors:** Eduardo Bernal, Francesca Rotondo, Veronica Roman-Reyna, Taylor Klass, Sujan Timilsina, Gerald V. Minsavage, Fernanda Iruegas-Bocardo, Erica M. Goss, Jeffrey B. Jones, Jonathan M. Jacobs, Sally A. Miller, David M. Francis

**Affiliations:** ^1^Department of Horticulture and Crop Science, College of Food, Agricultural, and Environmental Sciences, The Ohio State University, Wooster, OH, United States; ^2^Department of Plant Pathology, College of Food, Agricultural, and Environmental Sciences, The Ohio State University, Wooster, OH, United States; ^3^Department of Plant Pathology, College of Food, Agricultural, and Environmental Sciences, The Ohio State University, Columbus, OH, United States; ^4^Infectious Diseases Institute, The Ohio State University, Columbus, OH, United States; ^5^Department of Plant Pathology, Institute of Food and Agricultural Sciences, University of Florida, Gainesville, FL, United States; ^6^Emerging Pathogens Institute, University of Florida, Gainesville, FL, United States

**Keywords:** bacterial spot, *X. perforans*, tomato, whole genome sequencing, overwintering, clonal populations, migration, phylogenomic analysis

## Abstract

Changes in *Xanthomonas* race and species composition causing bacterial spot of tomato have occurred throughout the world and are often associated with epidemics. Knowledge of bacterial population structure is key for resistance discovery and deployment. We surveyed *Xanthomonas* spp. composition from processing tomato fields in the Midwestern United States over a 4-year period between 2017 and 2020, compared these to strains collected previously, and found that *X. perforans* is currently the most prevalent species. We characterized 564 *X. perforans* isolates for sequence variation in *avrXv3* to distinguish between race T3 and T4 and validated race designation using hypersensitive response (HR) assays for 106 isolates. Race T4 accounted for over 95% of *X. perforans* isolates collected in the Midwest between 2017 and 2020. Whole genome sequencing, Average Nucleotide Identity (ANI) analysis, core genome alignment and single nucleotide polymorphism (SNP) detection relative to a reference strain, and phylogenomic analysis suggest that the majority of Midwestern *X. perforans* strains collected between 2017 and 2020 were nearly identical, with greater than 99.99% ANI to *X. perforans* isolates collected from Collier County, Florida in 2012. These isolates shared a common SNP variant resulting an a premature stop codon in *avrXv3.* One sequenced isolate was identified with a deletion of *avrXv3* and shared 99.99% ANI with a strain collected in Collier Co., Florida in 2006. A population shift to *X. perforans* T4 occurred in the absence of widely deployed resistance, with only 7% of tomato varieties tested having the resistant allele at the *Xv3*/*Rx-4* locus. The persistence of nearly identical strains over multiple years suggests that migration led to the establishment of an endemic population. Our findings validate a genomics-based framework to track shifts in *X. perforans* populations due to migration, mutation, drift, or selection based on comparisons to 146 genomes.

## Introduction

*Xanthomonas* spp. causing the disease bacterial spot of tomato (BST) are not restrained by geographical location, and pathogen populations continue to change over time. In recent years *X. perforans* has dominated many tomato growing regions of the United States. More specifically, *X. perforans* race T4 (T4) was first observed in 1998, and since 2006 it has been dominant in Florida (Horvath et al., 2012; Timilsina et al., 2016). In the Midwestern United States multiple species of *Xanthomonas* have persisted, with the abundance changing over time (Sahin, 1997; Ma et al., 2011; Bernal, 2020). Between 1994 and 1996 collections in Ohio of xanthomonads causing BST showed that 51% of strains were characterized as race T1 (now recognized as *X. euvesicatoria*), 38% as T2 (*X. vesicatoria*), and 11% as T3 (*X. perforans*) (T3). Strains collected between 1994 and 1996 resulted in the first report of the presence of T3 in Ohio (Sahin, 1997). Collections between 2010 and 2013 showed a shift in the *Xanthomonas* species composition in Ohio and Michigan. Seventy percent of isolates were *X. hortorum* pv. *gardneri* (no race designation), 19% were *X. perforans*, and 11% were *X. euvesicatoria* (Ma et al., 2011; Ma, 2015). Between 2016 and 2017, collections in Indiana and North Carolina determined that *X. perforans* race T4 has become the dominant species followed by *X. hortorum* pv. *gardneri* and *X. euvesicatoria* (Egel et al., 2018; Adhikari et al., 2019).

The T3 and T4 race designation for *X. perforans* is based on the presence of a hypersensitive response (HR) when the pathogen protein AVRXV3 is recognized by the tomato locus *Xv3/Rx4* which corresponds to Solyc11g069020 at 53,579,795–53,583,729 bp on chromosome 11 (Robbins et al., 2009; Wang et al., 2011; Pei et al., 2012; Zhang et al., 2021). The resistant allele for *Xv3/Rx4* is found in multiple tomato accessions, including H7981, PI 126932, and PI 128216 (Scott et al., 1995, 1996; Pei et al., 2012; Zhang et al., 2021). If the tomato accession carrying *Xv3/Rx4* reacts with a HR after syringe inoculation with *X. perforans*, the strain is designated T3. If the strain fails to elicit an HR on plants with *Xv3/Rx4* it is designated T4 or T2. The T4 designation also implies an HR on tomatoes carrying the RXopJ4 locus from LA0716 (Sharlach et al., 2013). Several variants have been identified in *avrXv3* resulting in a loss of HR (Timilsina et al., 2016). These variants include a single nucleotide polymorphism (SNP) leading to an early stop codon, an insertion/deletion (indel) upstream of the coding sequence creating a “pseudogene/indel,” contig breaks in the coding sequence causing a truncation of the protein, or complete loss of the gene (Timilsina et al., 2016). Identification of *X. perforans* race T4 can therefore be characterized through plant-based assays using HR or sequence characterization of the bacterial effector.

In addition to effector mediated phenotypes, multi locus sequence alignment (MLSA) and whole genome sequencing have been used as tools to estimate phylogeny of *Xanthomonas* spp. (Timilsina et al., 2015, 2019). Whole genome sequencing has become a standard approach to measure the genomic similarity within and between species and to elucidate the predominant evolutionary forces governing population shifts of plant pathogens. Average nucleotide identity (ANI) uses a Basic Local Alignment Search Technique (BLAST) to scan 1 Kb fragments shared between contigs (or complete genomes). Estimates of the nucleotide identity from the individual shared fragments are then used to calculate the ANI across the genome (Goris et al., 2007; Figueras et al., 2014). Genomic variation can provide insight into pathogen dissemination across global geographic regions or within local agricultural production systems (Vinatzer et al., 2014; Monteil et al., 2016; Klein-Gordon et al., 2020, 2021; Perez-Quintero et al., 2020).

We collected *Xanthomonas* spp. from processing tomato fields in multiple counties in Ohio and Indiana between 2017 and 2020. Fruits were sampled at different stages of maturity, representing infection events that could occur 5–7 weeks apart. We sampled tomato varieties from various seed producers and fields of multiple growers established from seedlings grown in different transplant houses. Hypersensitive response assays, PCR amplification, and sequence characterization of *avrXv3*, *avrXv4*, and *avrBsT* were used for *X. perforans* race identification. Whole genome sequence data were used to assess the relationship between 38 *X*. *perforans* strains collected in the Midwest and 108 strains isolated from other geographical regions. The specific objectives of this study were to gain insight into the forces shaping pathogen populations by (i) describing the race composition of *X. perforans* in the Midwestern United States; (ii) identifying variants present in the *avrXv3* gene; and (iii) estimating the genomic relatedeness between *X. perforans* strains from Midwestern states and strains found in other geographical locations using phylogenomic approaches; and (iv) evaluating commercial tomato varieties for the presence of the *Xv3/Rx4* locus to determine the extent of resistance deployment in the region.

## Materials and Methods

### Sampling Tomatoes With Bacterial Spot Lesions

Sampling of *Xanthomonas* populations was performed in tomato fields in 2017, 2018, 2019, and 2020. For the first 3 years of collection a hierarchical strategy was employed. Field locations were organized according to state, counties, and regions within state that correspond to specific processing plants. Agricultural staff for major Midwestern tomato processors were contacted for field locations and field maps. Fruits with lesions were collected from fields in Ohio (Putnam, Hancock, Wood, Ottawa, Erie, Sandusky, and Seneca) and Indiana (Madison and Tipton) counties. These counties were divided into four sub-regions (North Central, Northwest, West Central OH and Central, IN). Growers in each sub-region sourced transplants from the same regional greenhouse grower(s) and sent harvested fruit to the same regional processing plants. In each field, fruit were sampled on a transect, with multiple green and red fruit collected per variety and per field. Fruit were processed in the Vegetable Pathology Lab, Department of Plant Pathology at The Ohio State University, Wooster, OH, United States using standard procedures previously described (Ma et al., 2011). A single isolate was maintained per lesion and given a numerical strain designation. In 2020, we isolated a few strains for the purpose of whole genome sequencing, described further below.

### Identification via BOX-PCR and qPCR

BOX-PCR and Real-Time PCR (qPCR) were used to discriminate between *X. perforans*, *X. euvesicatoria*, and *X. hortorum* pv. *gardneri*. BOX-PCR (BOX A 1R primer) was used to compare DNA banding profiles of the palindromic intergenic repetitive BOX A subunits conserved in bacterial species (Louws et al., 1994; Ma et al., 2011). All isolates were screened via a multiplex qPCR amplification of *hrpB7* as described in Strayer et al. (2016) to compare both strain discrimination methods and as a more robust species identification. For this study, we focused on further characterization of strains identified as *X. perforans*. The species level survey is described in a previous study (Bernal, 2020).

### Plant Material

Tomato line Ohio 88119 (Berry et al., 1995) and its near-isogenic sibling OH813A, having *Xv3/Rx4* (Bernal et al., 2020; Bernal and Francis, 2021) were used as susceptible and resistant controls to distinguish between *X. perforans* race T3 and T4 strains in all experiments. In addition, we tested isolates for HR against LA0716 as a control for *RXopJ4* interaction with the bacterial effector *avrXv4*. Tomato plants were grown in 1-gallon pots in a greenhouse managed by the Department of Horticulture and Crop Science at The Ohio State University, Wooster, OH, United States. The greenhouse temperature fluctuated between 22 and 27°C. The relative humidity was maintained between 50 and 70%.

Fresh-market and processing tomato varieties commonly used in the Midwestern United States were tested for the presence of the candidate gene for the *Xv3/Rx4* resistance locus. These include fresh-market varieties Camaro, Camp, FL91, FL47, Red Bounty, Southern Ripe, Grand Marshall, Bounty, Mariana, Southern Ripe and Tasti-Lee. Processing varieties were from OSU (Ohio and OX), Red Gold (GEM), Heinz Seed (H), Harris-Moran Seed (HM), Seminis Vegetable Seeds (Peto), Rispins (R), and Tomato Solutions (TSH) and included Ohio 8611, OX325, GEM 111, GEM183, GEM818, H1015, H101A, H1301, H1648, H1765, H1766, H2206, H3406, H3602, H5108, H7222, H8504, H9144, H9364, and H9706. H9996, H9997, HM1823, HM1892, HM3887, HM5900 HM8901, HM9903, Peto 696, R522695, TSH04, TSH05, TSH12, TSH18, TSH20, and TSH40. We used the marker PCC12 to indicate the presence of the *Xv3/Rx4* locus as described below (Pei et al., 2012).

### Inoculum Preparation and Hypersensitive Response Evaluation

Bacterial suspensions were prepared from 48 h-old culture plates initiated from single colonies and maintained at 28°C in darkness on yeast dextrose calcium carbonate (YDC) agar. Bacterial suspensions were prepared in sterile deionized water, and the optical density was adjusted to 0.15 optical density (OD) at an absorbance of 600 nm (∼10^7^ CFU/ml). Hypersensitive response (HR) leaf assays were performed within 2 h following the preparation of the suspension. The HR phenotype was evaluated to identify the race of 106 *X. perforans* strains and two positive controls, SM761 (T3) and Scott-1 (T4). This subset of strains comprised isolates from 2012 (16), 2013 (4), 2017 (29), 2018 (42), 2019 (15).

For evaluation of HR, inoculations were performed by leaf infiltration of 8–10-week-old potted plants of OH88119, OH813A, and LA0716 in the Williams Hall greenhouse, Wooster, OH. The plants were placed in a completely randomized design throughout the greenhouse. Bacterial suspensions were infiltrated into the underside of fully expanded leaflets using a 1-ml syringe (Robbins et al., 2009). The area of infiltration was 1–2 cm in diameter. Leaflets were inoculated between 10:00 AM-12:00 PM, and the HR phenotype was visually inspected and recorded daily for 5 days post inoculation (dpi). Five independent inoculation experiments were conducted between 2017 and 2019. Approximately 25 strains were evaluated at each time, with the addition of race T3 and T4 controls (Sahin, 1997; Timilsina et al., 2016). Three plants for each tomato genotype were syringe-inoculated with each strain to observe repeatability of HR symptoms. Inoculated leaflets were given a score of 1 = HR phenotype (confluent necrosis), 2 = susceptible response (necrosis expansion beyond inoculation area), 3 = no symptom development or unclear HR. In most cases, HR symptoms were most noticeable after 72 h. Strains that displayed the same phenotypic response in all biological reps (*n* = 3) within genotype were scored. Strains that displayed differences among the three replicates were re-tested.

### Bacterial and Plant DNA Isolation

DNA from bacterial isolates was extracted using the boiling method (Abrahamian et al., 2018) modified by the addition of a chloroform extraction. Single colony glycerol stocks were streaked and grown on nutrient broth yeast (NBY) medium for 48–72 h. Bacteria from a single colony was transferred into a 1.5 ml microcentrifuge tube and re-suspended in 400 ml of sterile water. Tubes were boiled at 100°C for 15 min in a dry-bath (Thermo Fisher Scientific, Waltham, MA, United States), and then immediately placed on ice for 5 min followed by centrifugation at 10,000 rpm for 5 min. An aliquot of 100 μL of isolated DNA was mixed with 300 μL of Tris EDTA buffer [10 mM Tris, 0.1 mM EDTA], and 400 μL of chloroform isoamyl alcohol (24:1) followed by centrifugation at 5,000 × *g* for 15 min. About 150 μL of the aqueous phase was transferred to a 96-well round bottom microtiter plate (Corning Inc., Kennebunk, ME, United States) containing 15 μL of sodium acetate (3M, at pH 5.3) and 330 μL of 70% ethanol followed by centrifugation at 5,000 × *g* for 10 min for DNA precipitation. Plates were air-dried and 100 μL of ddH_2_0 was added. For DNA isolation of plant material we used methods described in Bernal et al. (2020).

### Molecular Marker Analysis of *X. perforans* Races T3 and T4

Primers were designed to amplify *avrXv3* based on an alignment of wild-type and known variant alleles using the Geneious Prime software (Kearse et al., 2012). The reverse primer, avrXv3R, 5′-TGAGCGAGAGCTACTATCGCCTCC-3′ was previously described (Timilsina et al., 2016). The forward primer, avrXv3ef1, 5′-GGAAGCTTGGATTAAAGGGG-3′ was developed for this study such that the entire gene and known variants would be amplified. These primers flank *avrXv3* with a predicted amplicon of 358 bp, which spans a known SNP, and a 30 base indel upstream of the gene (Timilsina et al., 2019). All amplifications included controls, T3, T4, *X. euvesicatoria* and *X. hortorum* pv. *gardneri*. The *avrXv3* gene was PCR-amplified in 564 *X. perforans* strains to determine the allele associated with the HR phenotype. Polymerase Chain Reaction (PCR) was conducted in 20 μL reactions consisting of 2 μL of Buffer A [10 mM Tris–HCL, 50 mM KCl, 1.5 mM MgCl_2_,], 0.8 μL of 1.25 mM dNTP, 0.2 μL of 10 μM forward and reverse primer, 12.4 μL of ddH_2_0, 0.4 units of *Taq* DNA polymerase, and 4 μL of DNA template using the following cycling conditions: (1) 94°C for 3 min, (2) 94°C for 1 min, (3) 63°C for 30 s, (4) 72°C for 1 min, (5) 72°C for 5 min. Steps 2–4 were repeated 35 times prior to the final annealing step. Amplicons were evaluated based on restriction digestion and/or polymorphism detected based on agarose gel electrophoresis. The presence of the known SNP205nt was evaluated using the restriction enzyme *Alu*I (New England Biolabs Incorporated, Ipswich, MA, United States) in a cleaved amplified polymorphic (CAP) detection approach. PCR products were digested by combining 16 μl of PCR product with 1.0 μL of 1X CutSmart^®^ Buffer (New England Biolabs Incorporated, Ipswich, MA, United States), 0.4 μL of *Alu*I (10,000 units/ml), and 2.6 μL of ddH_2_0 for 3 h. Amplicons were separated on a 2% agarose gel at 180V for 1 h and 30 min and detected using ethidium bromide fluorescence.

### Whole Genome Sequencing of *X. perforans* Strains

In total, 38 *X. perforans* strains from the Midwestern United States were selected for whole genome sequencing. The criteria for selecting a subset from the 564 strains for whole genome sequencing was based on (1) year of isolation, (2) location, (3) allele at the *avrXv3* locus, and (4) HR response (Supplementary Table 1). Strain SM1852 was included as a representative of those with a failure to amplify using the *avrXv3* primers. Isolates were selected from clean single colony cultures stored in glycerol for DNA extraction. A total of 14 unique strains were sequenced using an Illumina MiSeq System (Illumina Inc.) by University of Florida. Reads were assembled using SPAdes v.3.11 (Bankevich et al., 2012). A detailed pipeline for assembly and filtering process of genomes was described previously (Timilsina et al., 2019). The remaining 24 unique *X. perforans* strains were sequenced using an Illumina iSeq 100 System with the NextEra DNA Flex Library Prep protocol. One strain, SM1852-18 was sequenced three times over two sequence runs. The same glycerol stock for SM1852-18 was used in all cases. In the first run (R1) an independent single colony was used as a source of DNA. We consider this to be a biological replicate. In the second run, a different colony was selected, and the same DNA was used with two different sets of adapters (R2 and R3). We consider these to be technical replicates. This process of sequencing the same DNA was conducted as a control to determine error rates. FastQC (Andrews, 2010) was used for quality control of the raw sequence data. Reads were assembled using SPAdes v.3.14.1 (Bankevich et al., 2012). Reads and assemblies are available through the National Center for Biotechnology Information (NCBI) under BioProject accession PRJNA795842. These assemblies were annotated using the NCBI Prokaryotic Genome Annotation Pipeline (PGAG).

### Characterization of *avrXv3* Alleles and Presence of *avrBsT* and *avrXv4*

A total of 108 sequences of strains available in public databases were narrowed to 24. The criteria for selecting the subset of 24 from the 108 publicly available sequences was (1) location, (2) year of isolation, (3) allele at the *avrXv3* locus, and (4) previously defined *X. perforans* clade or group (Schwartz et al., 2015; Newberry et al., 2019; Timilsina et al., 2019). Passport data that emphasize these criteria are included in Supplementary Table 1. For the analysis presented here, the genomes of 64 *X. perforans* strains, 38 strains collected between 2017 and 2020 in the Midwest, two biological and technical replicates, and 24 sequences of bacterial strains available in public databases were utilized to characterize alleles of *avrXv3*, *avrBsT*, and *avrXv4*. A 1.5 kb region including the *avrXv3* gene from *X. perforans* strain CFBP7293 (NZ_MOLQ01000001.1) was extracted and searched in a custom database containing the 64 *X. perforans* genome sequences formatted as FASTA files. The FASTA sequences were then formatted as a Basic Local Alignment Search Tool (BLAST) database and searched using BLASTN with default settings in the Geneious Prime software (Altschul et al., 1990; Kearse et al., 2012). The 1.5 kb region from all strains was aligned and mapped to CFBP 7293 using Multiple Sequence Comparison by Log-Expectation (MUSCLE) version 3.8.425 (Edgar, 2004). To further characterize novel *avrXv3* alleles we utilized Burrows-Wheeler aligner (BWA), BWA-MEM algorithm, v.0.7.17 (Li, 2013) to map the raw reads to *avrXv3* using the reference T3 strain Xp91-118. SAMtools v1.13 (Li et al., 2009) was utilized for post-processing of alignments. The Integrative Genomics Viewer (IGV) v2.8.13 (Robinson et al., 2011) was utilized to visualize mapped reads of sequenced strains using the “show read pairs” option. To determine the presence of *avrBsT* and *avrXv4* we extracted the gene sequences from *X. perforans* strain TB6 contig15 (NZ_JZWA01000015.1) and *X. perforans* strain NC373 (MK583029.1), respectively. This sequence was used for BLAST and MUSCLE alignment as described for *avrXv3*.

### Average Nucleotide Identity Pairwise Analysis

We performed an average nucleotide identity (ANI) pairwise analyses to determine whole genome sequence variation between 24 reference strains, 38 *X. perforans* strains sequenced from our 2017 to 2020 collection, and two controls. ANI for *X. perforans* strains was completed using the enviomics toolbox ANI calculator from the Kostas lab (Rodriguez-R and Konstantinidis, 2016). The calculator estimates the ANI between genomes by comparing 1 kb intervals (Goris et al., 2007). The approach uses reciprocal best matches as a way to measure genetic relatedness. The calculator uses the BLASTN algorithm with X = 150 (where X is the drop-off value for gapped alignment), *q* = 21 (where q is the penalty for nucleotide mismatch) and F = F (where F is the filter for repeated sequences) (Goris et al., 2007). The pairwise ANI analysis quantifies genome wide identity based on SNPs and gaps between strains.

### Core Gene Analysis

A core-gene phylogenetic tree for *39 X. perforans* strains was created to assess the diversity within strains. Five housekeeping genes *lepA*, *gyrB*, *lacF*, *gapA*, and *gltA* were identified in these 39 strains and concatenated (Timilsina et al., 2015). The sequences of housekeeping gene accessions were extracted from NCBI as follows: *lepA* (KM492599.1), *gyrB* (KM492331.1), *lacF* (KM492465.1), *gapA* (KM492063.1), and *gltA* (KM492197.1). Sequences identified with the Basic Local Alignment Search Tool, BLASTN, in the Geneious Prime software (Altschul et al., 1990; Kearse et al., 2012). The phylogenetic tree was developed using the Geneious tree builder (Kearse et al., 2012). Standard parameters were set using a global alignment with free end gaps, a cost matrix of 65% similarity, Tamura-Nei genetics distance model, and a neighbor-joining tree method. For the pair-wise alignment option for building the distance matrix a gap open penalty and gap extension penalty of 12 and 3 were used, respectively.

### Phylogenomic Analysis

Phylogenomic relatedness between *X. perforans* strains was assessed using a whole-genome SNP genotyping alignment method. The Harvest suit package, Parsnp v.1.2 (Treangen et al., 2014) utilizes whole-genome alignment, read mapping, and k-mer analyses for SNP identification. The Parsnp output produces a core genome alignment, SNP variant calls, gap analysis, and a phylogenomic tree (Treangen et al., 2014). Parsnp was used to align all 64 *X. perforans* genomes (including strain replicates) to Xp91-118 reference T3 strain. The sequence for Xp91-118 is the most complete genome for *X. perforans* and is extensively used as a reference (Abrahamian et al., 2019). Alignments were implemented using the log-expectation function in MUSCLE with trees rooted to Xp91-118 based on maximum-likelihood nearest-neighbor interchanges as implemented in FastTree2 (Price et al., 2010). Gingr v1.3, a graphic user interface from the Harvest suit package, was used to visualize the phylogenomic tree. The newick formatted text file was extracted from Gingr v.1.3 and the Interactive Tree of Life (iTOL) v5 (Letunic and Bork, 2021) online tool was used to display and annotate the phylogenomic tree. The Parsnp variant call output consisted of 23,203 SNPs that were used to determine experimental error and to assess polymorphism between and among strains.

Experimental error was determined by comparing SNPs between the SM1852-18 control strain sequenced three times (SM1852-R1, SM1852-R2, and SM1852-R3). The SNP comparison for the three controls was used to set a baseline for error that is a composite of sequencing error and assembly error but will not reflect systematic errors in assembly. This error rate was estimated based on the summation of all SNPs between technical replicates divided by the size of the reference genome (4,898,349 bp), Xp91-118 used in the alignment. Strains displaying a SNP polymorphism rate lower than or within the range detected for technical resequencing of control strain replicates were regarded as identical within the resolution of our methods.

### Decay of Linkage Disequilibrium

Analysis of LD was performed using the R package “sommer” (Covarrubias-Pazaran, 2016, 2018). The matrix of 23,203 SNPs was extracted from the Parsnp variant call output and transformed such that accession sequences were row names and SNP (locus) were column headings. The SNP genotyping matrix consisted of “0” for reference genome and “1” for alternative allele calls. The locus name and position were extracted and combined with a vector for linkage group = 1, and this matrix provided the “map” file for analysis using the LD.decay function.

## Results

### Changes in *X. perforans* Race Distribution

**FIGURE 1:**
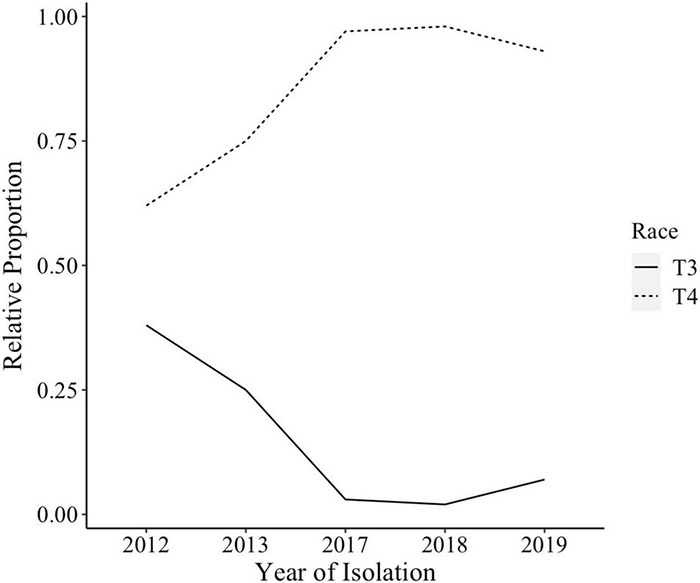
*Xanthomonas perforans* race composition for strains isolated from tomatoes between 2012 and 2019. *X. perforans* strains isolated in 2012, 2013, 2017, 2018, and 2019 were screened using hypersensitive response (HR) assay to determine their race. By 2017, 93% of all *X. perforans* strains were categorized as race T4 based on hypersensitive response and PCR amplification of *avrXv3*.

**FIGURE 2:**
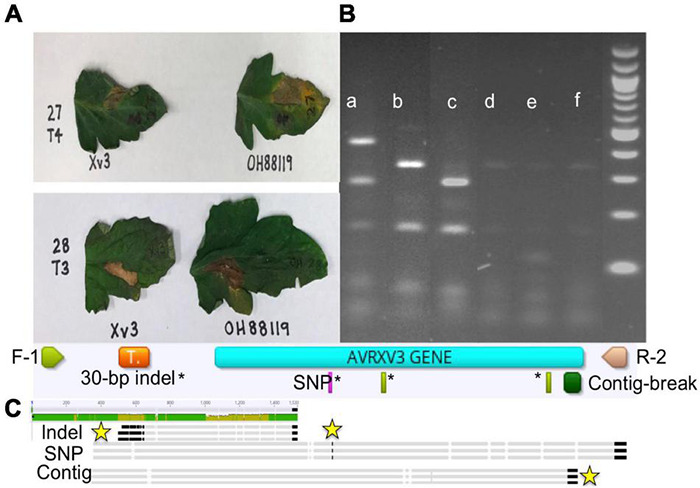
Hypersensitive response (HR), genotype banding patterns for avrXv3 mutations, and graphical presentation of known AvrXv3 mutations. **(A)** Xv3 OH813A with Xv3/Rx4 inoculation elicits an HR in the presence of *Xanthomonas perforans* race T3 (lower panel) and not T4 (upper panel). OH88119 was utilized as a control to show susceptibility to both races. **(B)** Shows PCR amplification and banding patterns of *X. perforans* race T4 mutations and race T3. (a) *X. perforans* race T4 (Scott-1) digest band size 496, 280, and 164 bp, (b) *X. perforans* race T3 (SM761) digest band sizes 332 and 164 bp, (c) T4-SNP (1828-17) digest band sizes 280 and 164 bp (d,e) *X. euvesicatoria* (110C and 767), (f) *X. hortorum* pv. *gardneri* (SM775-12) show weak bands due to non-specific amplification as those *Xanthomonas* species do not possess *avrXv3*. A 1 kb ladder (far-right) was used as a size standard, each band represents 100 bp. **(C)** F-1 and R-2 refers to the flanking primers used to amplify avrXv3. The orange box represents the location of a previously known pseudogene. The pink rectangle highlights the location of a known SNP mutation, which also corresponds to a restriction site for *Alu*I. The green rectangles* represent other cut sites for *Alu*I. The large dark green box represents the location of a known mutation creating a contig break. Sequence alignment of *X. perforans* strains with different mutations is shown at the end. The yellow stars mark the location of the mutations.

The species makeup of *Xanthomonas* strains collected between 2017 and 2020 in the Midwest has changed relative to previous reports (Sahin, 1997; Ma et al., 2011; Ma, 2015). *X. perforans* T4 now appears to be the predominant species and race based on hypersensitive response assays, and sequence analyses of *avrXv3* (Bernal, 2020; Rotondo et al., 2022). In 2012 *X. perforans* race composition was approximately 40% T3 and 60% race T4. Since 2017, race T4 has accounted for over 93% of isolated *X. perforans* strains (Figure 1). The tomato genotype OH813A displayed a clear HR when inoculated with a race T3 control strain, SM761, and water-soaked necrosis when inoculated with the T4 control strain, Scott-1. In all assays OH88119 was susceptible to both control strains and no HR was observed (Figure 2A). LA0716 displayed HR when inoculated with control strains. Out of 106 strains tested >95% were identified as race T4 based on HR response to tomatoes carrying *Xv3/Rx4* and *RXoJ4*.

We screened 564 *X. perforans* strains using PCR amplification and identified a known SNP variant within *avrXv3* with a high frequency (94%). This SNP was previously described at the 205 nucleotide position in *avrXv3* (Klein-Gordon et al., 2021), and was only found in isolates collected between 2017 and 2020 in the Midwest. No amplification of *avrXv3* was observed for *X. euvesicatoria* and *X. hortorum* pv. *gardneri* controls as expected, though faint bands that we ascribe to non-specific amplification were observed (Figure 2B). Several strains collected between 2010 and 2013 contained a previously described T4-Contig variant (Klein-Gordon et al., 2021). For thirty seven *X. perforans* strains (∼6.5%) *avrXv3* was not amplified. One of these strains, SM1852-18, was used for whole genome sequence and we detected a ∼4 Kb deletion which extended beyond. The coding sequence of *avrXv3* (see section “Characterization of *X. perforans* Strain SM1852-18 Lacking *avrXv3* and *avrBsT*” below). We did not detect the presence of novel insertion deletion variants or mutations within the coding sequence of *avrXv3* (Figure 2C). The presence of the previously characterized SNP205nt variant and a lack of other variation was verified using Sanger sequencing of amplicons (data not shown). There was a strong association between the HR phenotype and the PCR-based genotypic screening of *avrXv3*, with 101 of 106 strains showing complete agreement. Five strains were phenotypically classified as T3 but displayed the SNP205nt variant in the *avrXv3* locus.

### Tomato Germplasm

Forty-three commercial varieties were tested for the presence for the *Xv3/Rx4* allele conferring HR to *X*. *perforans* race T3. Heinz varieties H2206, H5108, and H9997 and the Harris-Moran variety HM6900 had the *Xv3/Rx4* allele associated with HR in the presence of *avrXv3*. H5108, an early season hybrid, accounts for less than 7% of the processing tomato acreage in Ohio and Indiana. Varieties H2206 and H9997 are considered experimental and are not widely used. None of the fresh-market varieties tested possessed the *Xv3/Rx4* allele associated with HR.

### Whole Genome Sequence Analyses of *X. perforans* Strains

Sequence reads and assemblies are available through NCBI Bioproject PRJNA795842. Sequences generated through iSeq ranged from 6x to 10x coverage while sequences generated on the MiSeq ranged from 13x to 17x. Biological and technical replication was used to estimate an experimental error as determined by resequencing and reassembly prior to phylogenomic analysis (Table 1). This error estimate quantifies the number of single-nucleotide differences between replications and accounts for sequence and assembly error but will not account for systematic errors due to methodologies nor the potential for mutations between single colony isolates. We refer to experimental error in this context. A total of 23,203 SNPs were extracted from Parsnp to characterize the genomic relatedness between *X. perforans* strains. SNPs between specific *X. perforans* strains were calculated relative to the whole genome size (4,898,349 bp) of *X. perforans* strain 91–118 (Table 1). The allele mismatch between technical reps, SM1852-R1 (run 1, rep 1), R2 (run 2, rep 2), and R3 (run 2, rep 3) was between 23 and 67 SNPs. Comparison of SM1852-R2 vs. SM1852-R3 showed a difference of 50 SNP polymorphisms with an allele mismatch of 1.02 × 10^–5^ relative to the whole genome (Table 1).

**TABLE 1:** Pairwise analysis of polymorphism between technical replicates, biological replicates, and strains.

Comparison 1				Comparison 2							
Isolate^a^	Xp Group^b^	Location	Year	Isolate	Xp Group	Location	Year	SNP^c^ no.	SNP^d^ mismatch	GAP^e^ analyses	CDS^f^ diff.
SM1852-R1	Group 1B (SC2)	OH	2018	SM1852-R2	Group 1B (SC2)	OH	2018	67	1.37E-05	0.79%	58
SM1852-R1	Group 1B (SC2)	OH	2018	SM1852-R3	Group 1B (SC2)	OH	2018	23	4.70E-06	0.15%	72
SM1852-R2	Group 1B (SC2)	OH	2018	SM1852-R3	Group 1B (SC2)	OH	2018	50	1.02E-05	0.45%	14
Xp17-12	Group 1B (SC2)	FL	2006	SM1852-R1	Group 1B (SC2)	OH	2018	28	5.72E-06	0.41%	92
GEV893	Group 1A (SC1)	FL	2012	GEV909	Group 1A (SC1)	FL	2012	9	1.84E-06	0.20%	29
GEV893	Group 1A (SC1)	FL	2012	SM1806	Group 1A (SC1)	OH	2017	9	1.84E-06	0.16%	76
GEV893	Group 1A (SC1)	FL	2012	SM1856	Group 1A (SC1)	OH	2018	46	9.39E-06	0.14%	77
GEV893	Group 1A (SC1)	FL	2012	SM234	Group 1A (SC1)	OH	2019	60	1.22E-05	0.12%	86
GEV893	Group 1A (SC1)	FL	2012	SM176	Group 1A (SC1)	OH	2020	64	1.31E-05	0.16%	80
SM1843	Group 1A (SC1)	OH	2017	SM1011	Group 2 (SC3)	OH	2013	5341	1.09E-03	3.01%	318
SM1807	Group 1A (SC1)	IN	2017	SM1809	Group 1A (SC1)	OH	2017	46	9.39E-06	0.08%	8
SM1807	Group 1A (SC1)	IN	2017	GEV893	Group 1A (SC1)	FL	2017	9	1.84E-06	0.20%	75
SM1809	Group 1A (SC1)	OH	2017	GEV893	Group 1A (SC1)	FL	2017	53	1.08E-05	0.10%	79
SM1807	Group 1A (SC1)	IN	2017	SM779	Group 2 (SC3)	OH	2012	5279	1.08E-03	3.01%	581
SM1809	Group 1A (SC1)	OH	2017	SM779	Group 2 (SC3)	OH	2012	5323	1.09E-03	3.03%	577
GEV893	Group 1A (SC1)	FL	2012	SM779	Group 2 (SC3)	OH	2012	5280	1.08E-03	2.95%	656

*^a^Isolate names are referenced in Supplementary Table 1. SM1852-R1 is a single colony isolate sequenced on the first run, SM1852-R2 and SM1852-R3 are from the same DNA source using separate sets of adapters during library preparation prior to sequencing.*

*^b^Xp Group refers to phylogenetic clustering described previously (Schwartz et al., 2015; Newberry et al., 2019; Timilsina et al., 2019).*

*^c^SNP no., the number of single nucleotide polymorphisms (SNPs) between two isolates.*

*^d^SNP mismatch, number of SNPs relative to whole genome size of Xp 91-118 (4,898,349 bp).*

*^e^GAP analyses, percent of total unaligned sequences displaying gaps in pairwise comparisons with ANI.*

*^f^CDS diff., pairwise comparison of the number of coding sequences derived from the NCBI Prokaryotic Genome Annotation Pipeline (PGAG).*

After establishing an error based on resequencing and reassembly, we characterized the allele mismatch between reference strains and sequenced isolates collected from Midwest between 2017 and 2020. The sequences for isolates GEV893 and GEV909 from Collier County, FL displayed an allele mismatch of 1.84 × 10^–6^. Comparisons between GEV893 and strains from the Midwest displayed a similar, or lower, allele mismatch relative to the SM1852-R1, R2, and R3 controls (Table 1). These mismatch estimates fall within our technical error and are consistent with expected mutation rates of genes per generation (Balin and Cascalho, 2010; Mhedbi-Hajri et al., 2013). These results provide evidence that these strains may be clonal based on their near identity.

Information extracted from the annotation of sequences provided comparative estimates of the number of coding sequences (CDS) while ANI provided pairwise SNP and alignment gap information (Table 1). Technical reps SM1852-18-R2, and SM1852-18-R3 have a difference of 14 CDS though the same DNA was used for both runs. SM1852-18 biological reps, R1 and R3, show a difference of 72 CDS. When comparing annotations of individuals collected between 2017 and 2020, we observe CDS differences lower than noted for our technical reps. For example, SM1807-17 and SM1809-17 have a difference of 8 CDS even though these were isolated in Indiana and Ohio, respectively. The ANI analysis provides another approach to assessing the potential for loss or gain of sequences based on open gap analysis. In comparing technical replicates R2 and R3 open gaps account for 0.45% of the genome. In comparing R1 and R2, open gaps accounting for 0.79% of the genome were found while comparisons of R1 and R3 reveal gaps accounting for 0.15%. Comparing strains known to differ in gene content, SM1807 with the SNP205nt variant of AvrXv3 and SM779 (contig), revealed gaps accounting for 3% of the genome. A comparison of SM1809 and GEV893 identified gaps accounting for only 0.1% of the genome. These results suggest gene-loss or addition are not accounting for large amounts of variation between genomes especially when comparing strains with near-identity based on SNP analysis.

### Phylogenomic Whole Genome Analyses of *X. perforans* Strains

A preliminary comparison of phylogenetic analysis was conducted using three measures of genome variation. Comparison of five core genes, genome wide ANI, and core genome alignment and SNP detection relative to a reference strain Xp91-118 displayed similar structure (Supplementary Figure 1). Within the Group 1A (SC1) clade, 16 group identically between methods. However, the core-gene tree contains an additional 7 strains which do not have the SNP205nt allele. These strains are BRIP62383, BRIP2389, BRIP62404, 91-118, Xp5-6, 4P1S2, and CFBP7293. The T4-Contig strains [Group 1B (SC2)] group identically between methods. ANI and whole-core-genome approaches offered higher resolution, likely due to the increase in sequence comparisons. Therefore a phylogenomic approach based on core genome alignment and pairwise ANI was utilized to evaluate diversity for a larger set of *X. perforans* sequenced strains.

**FIGURE 3:**
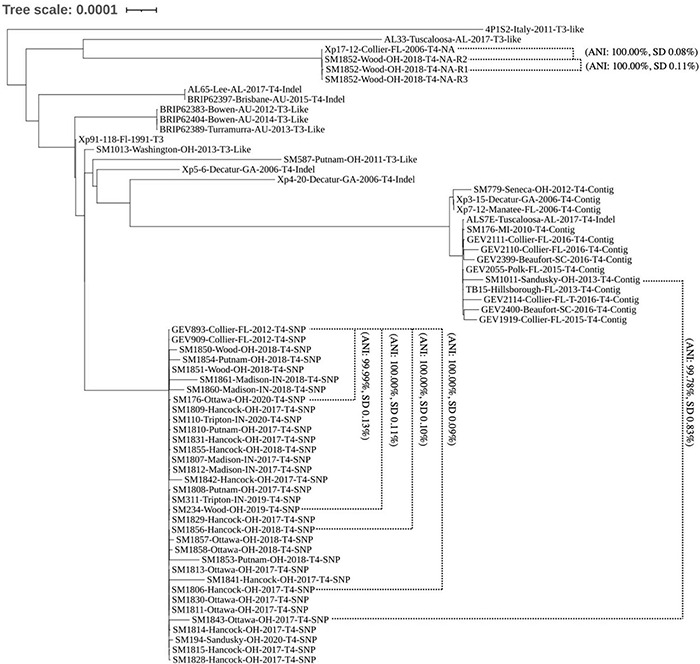
Phylogenomic tree of *X. perforans* isolates geographically distributed worldwide. Phylogenomic tree of 64 *Xanthomonas perforans* strains based on whole genome sequencing. Strains are denoted by the name, location of isolation, year, race-type, and known mutation in *avrXv3*. NA refers to the absence of *avrXv3.* Two-way-Average Nucleotide Identity values and standard deviations are denoted for specific comparisons between and within clades. The majority of strains isolated and sequenced in 2017–2020 are genetically identical. These strains group under the same clade as strains GEV909, GEV893 isolated from Florida in 2012. These strains all have the same SNP mutation in *avrXv3*.

To determine the genomic diversity across the 38 strains sequenced in this study, a subset of 24 publicly available genomes showing variation across *avrXv3* were selected for reference. These 24 genomes were selected from a larger data set, showing unique sequence variation in *avrXv3* previously described (Timilsina et al., 2016; Newberry et al., 2019). In addition, genomes were selected across time and space based on collection of the original strains to further investigate potential geographical stratification of *X. perforans* isolates. Strains sequenced from 2010, 2011, 2012, and 2013 were classified as T3-like or T4. The designation T3-like refers to strains that have a complete *avrXv3*, but have not been evaluated for HR. T3-like strains SM1013-13 and SM587-11 were more genetically similar to the reference strain Xp91-118 race T3 (Figure 3). Sequenced strains SM176-10, SM779-12, and SM1011-13 from 2010, 2012, and 2013, respectively, were collected when *X. hortorum* pv. *gardneri* was the predominant species in the Midwest (Ma et al., 2011; Ma, 2015) and contained a T4-Contig variant. This sequence variant creates a break in the coding sequence of *avrXv3*, similar to other strains from Florida. These strains clustered under Group 2 (Figure 3), previously described (Schwartz et al., 2015; Timilsina et al., 2019).

The majority of *X. perforans* strains isolated from the Midwest between 2017 and 2020 clustered within the same clade as Florida strains GEV893 and GEV909, isolated in 2012 (Figure 3). This genetic cluster has previously been described as Group 1A, or sequence cluster 1 (SC1) (Newberry et al., 2019; Timilsina et al., 2019). ANI was calculated between strains and clusters within the phylogeny to further quantify relationships. For example, the ANI between SM1852-R2 and SM1852-R3 was 100% SD 0.02 while SM1852-R1 and SM1852-R2 was 100% SD 0.11%. In contrast, Group 1A sequence cluster SC1 strain SM1843-17 and Group 2 strain SM1011-13 were 99.78% SD 0.83% identical based on ANI (Figure 3). Comparison of whole genome sequence for GEV893 and isolates from the Midwest collected between 2017 and 2020, including SM194-20 collected from a volunteer plant in a wheat field, displayed an ANI between 99.99% and 100.00% (Figure 3). These strains all had the SNP205nt *avrXv3* variant and the effector *avrBsT*. Phylogenetic comparison of GEV893 with strains from the Midwest isolated between 2017 and 2020 suggests that Group 1A cluster SC1 predominates and is likely clonal. Further evidence of clonality is suggested by a lack of LD decay across the entire genome (data not shown).

One strain, SM1852-18 did not cluster in Group 1A. All three technical replicates of strain SM1852-18-R1, R2, and R3 clustered with the strain Xp17-12 previously described (Schwartz et al., 2015; Abrahamian et al., 2019). Strain Xp17-12 was collected in Florida in 2006 and was nearly identical to SM1852-18-R2 based on ANI (100% SD 0.08%). The strains SM1852-18 and Xp17-12 do not contain *avrXv3* or *avrBsT* (described further below).

### Characterization of *X. perforans* Strain SM1852-18 Lacking *avrXv3* and *avrBsT*

**FIGURE 4:**
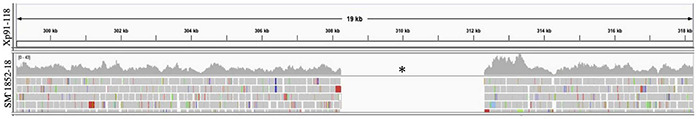
Mapping of sequence reads to *avrXv3* from Xp91-118. Burrows Wheeler Alignment was utilized to map reads *from X. perforans* isolate SM1852-18 to Xp91-118. Integrative Genome Viewer was utilized to visualize mapped reads. No reads (denoted with a black asterisk) from SM1852-18 mapped to Xp91-118.

SM1852-18 did not induce a hypersensitive response on OH813A and failed to amplify *avrXv3* in PCR, consistent with a deletion of *avrXv3*. Whole-genome sequence analyses of *X. perforans* strain SM1852-18 confirmed the absence of effectors *avrXv3* and *avrBsT*. A ∼4 Kb region encompassing *avrXv3* from Xp91-118 showed no reads mapped from SM1852-18 (Figure 4). The presence/absence of avrBsT was based on BLASTN searches and SM185218 lacked this effector as well (data not shown). All other sequenced strains classified as T4-SNP or T4-Contig contained both the effectors *avrBsT* and *avrXv4*. The presence of *avrBsT* was expected, as the effector is common in both Group 1a/SC1 and Group 2/SC3, previously described (Newberry et al., 2019).

## Discussion

Our results validate a genomics-based framework to track shifts in *X. perforans* populations due to migration, mutation, drift, or selection. We accessed 108 *X. perforans* whole-genome data sets from public sources and distilled these into 24 reference strains representing diversity within the species, major clades (Timilsina et al., 2019), date of collection, and geographic origin of the isolate. We evaluated 106 *X. perforans* strains using HR to distinguish between race T3 or T4 and we utilized PCR to amplify *avrXv3* in 564 Midwestern isolates and controls strains. Our results demonstrate that *X. perforans* race T4 is currently the predominant species and race in the Midwestern United States. These results parallel those reported for Florida, Indiana, and North Carolina (Timilsina et al., 2016; Egel et al., 2018; Adhikari et al., 2019; Klein-Gordon et al., 2020).

We observed a strong correlation between HR response and the PCR amplification assay for the *avrXv3* effector. The error rate of <5% could have resulted from a misclassification of HR in OH813A, despite repeated tests. However, it is also naïve to assume that loss-of-function SNPs and insertion/deletions are the sole reason for loss of HR. It is possible that regulatory mechanisms affecting expression and or modification of the *avrXv3* effector might also account for this observed variability associated with genetic characterization and HR phenotype.

We utilized whole-genome phylogenomic analyses to investigate sequence variation between the 24 reference strains and 38 newly sequenced genomes from the Midwestern United States. Based on the characterization of strains collected between 2017 and 2020, we determined that the most abundant clone (37 of 38 sequenced strains) belongs to the Group 1A/SC1 clade of *X. perforans*, contains a known SNP variant in *avrXv3* (SNP205nt), and both *avrBsT* and *avrXv4.* The strains collected between 2017 and 2020 differ from strains collected in the same region between 2010 and 2013 and show sequence identity, within the bounds of experimental error, with a strain collected in Florida previously. Although these analyses do not provide insight into how the Group 1A/SC1 clone was introduced, transplant shipment from the South, seed introduction, or weather patterns all offer possible routes. We also identified a strain, SM1852-18, lacking *avrXv3* and *avrBsT*. This strain and other isolates that failed to amplify *avrXv3* account for less than 1.2% of the current Midwestern *X. perforans* population. Phylogenetic analysis place SM1852-18 in the previously described Group 1B/SC2 clade. ANI demonstrated near identity to Xp17-12, isolated in Florida in 2006, suggesting a migration mechanism for even rare constituents of the population. This strain accounts for nearly 16% of 280 sequenced from Florida in recent years (Klein-Gordon et al., 2021). The sequence differences detected within strains classified as Group 1A/SC1 are consistent with known mutation rates for Xanthomonas which range from 10^–6^ to 10^–5^ base changes per generation (Mhedbi-Hajri et al., 2013) and are within the range of experimental error based on technical replication. The lack of sequence polymorphism between *X. perforans* strains collected across space and time, the lack of LD decay, and the phylogenetic clustering suggests an established clonal population. Clonal populations are defined by having two distinct features, high linkage disequilibrium (LD) and strong phylogenic signal (Tibayrenc and Ayala, 2012) consistent with the *X*. *perforans* strains analyzed here and suggests that the strains we characterized are descended from a single common ancestor that has been maintained for multiple years.

It is unlikely that Group 1A/SC1 is reintroduced each year on seed. Hybrid tomato seed tends to be produced in India, Thailand, and China with production locations varying by producer and current costs. It is unlikely that the diversity of producers, varieties, and seed lots sampled would all carry a single clone. If continuous migration through seed exchange was occurring every year, or if strains were moving with weather annually, we would expect to find greater variation in Midwestern states as new species and genotypes are introduced annually. The occurrence of the T4-SNP clone across varieties, regions, growers would seem to rule out an epidemic originating from contaminated seed and reestablishing each year. Xanthomonads surviving in transplant facilities could also explain the maintenance of clonal populations. More recent *X. perforans* population studies across Florida commercial tomato fields have shown that strains could be associated with tomato production systems and transplant facilities (Klein-Gordon et al., 2020, 2021). It is possible that the T4-SNP clone was introduced into the Midwestern United States via seed or transplants and is now surviving in transplant greenhouses, in fields, or on alternate hosts.

We identified a *X. perforans* T4-SNP Group 1A/SC1 isolate from a volunteer tomato plant found in a wheat field in 2020. An early description of bacterial spot suggested that *Xanthomonas* spp. were capable of overwintering in Indiana (Gardner and Kendrick, 1921). It was reported that volunteer tomato plants with bacterial spot symptoms were observed in a corn field, even though no tomato fields were planted nearby. The disease was present the year before, suggesting that the bacteria overwintered in debris or seed from the field (Gardner and Kendrick, 1921). In a different study it was shown that *X. vesicatoria* contributed 0.3% of the field soil microbial population in winter and spring sampling in Indiana, and was attributed to continuous inoculum from standing stalks of dead tomato plants (Peterson, 1963). Other studies have shown and/or suggested that xanthomonads can overwinter on wheat, weeds, and plant debris (Diachun and Valleau, 1946; Leben, 1981). Identification of T4-SNP Group 1A/SC1 isolates on volunteer tomato plants provides evidence of a possible overwintering of *X. perforans* in the Midwest.

Mutation in *avrXv3* does not appear to be driving population shifts in the absence of widespread resistance. The SNP205nt variant and the strain lacking *avrXv3* were first described in Florida strains in 2012 and 2006, respectively. Of 564 isolates analyzed, only two previously described *avrXv3* variants were identified between 2017 and 2020. Previous research demonstrated that some Ohio *X. perforans* race T3 strains characterized lack the effector *avrBsT* present in T4 strains (Timilsina et al., 2016). The effector *avrBsT* has been shown to provide a fitness advantage in the field compared to a knock-out recombinant (Abrahamian et al., 2018; Sharma et al., 2021). Therefore, the persistence of T4 may be related to fitness associated with a specific effector. We determined that all *X. perforans* T4-SNP strains had *avrBsT* with the exception of SM1852-18, similar to Xp17-12 isolated in Florida in 2006 (Timilsina et al., 2016).

Our results show that the race shift observed in the Midwestern United States is due to the introduction or migration of a strain of *X. perforans* possessing a previously described SNP205nt mutation in *avrXv3*. Given the low rate of *Rx-4*/*Xv3* deployment, it is unlikely that mutation in *avrXv3* was selected in response to the plant-pathogen arms race nor is resistance gene pressure responsible for maintaining this change. We cannot rule out multiple introductions, although it seems unlikely that the same clone would be introduced multiple years from multiple seed sources. The evidence based on amplification of *avrXv3*, whole genome sequencing, and analysis of isolates collected over several years suggests that the population of *X*. *perforans* T4 is largely descended from and is genetically identical to a single common ancestor that has been introduced and maintained for multiple years.

## Data Availability Statement

The datasets presented in this study can be found online as cited in the Supplementary Material. Sequences and annotation are available at NCBI under BioProject accession PRJNA795842.

## Author Contributions

DF developed the experimental design, sampled tomatoes with bacterial spot lesions throughout the Midwestern United States, and assisted with data analysis. EB established the experimental design, performed the bacterial inoculations, genotyping, phylogenomics, and strain characterization, and analyzed all data. FR and SAM isolated and genotyped the bacterial strains at the species level. FR cultured and prepared bacterial strains for inoculations. VR-R, TK, ST, GM, FI-B, JMJ, JBJ, and EG conducted library preparation and whole genome sequencing of strains isolated over the 4-year period, and provided guidance on tools and resources to conduct data analyses. All authors contributed to the article and approved the submitted version.

## Conflict of Interest

The authors declare that the research was conducted in the absence of any commercial or financial relationships that could be construed as a potential conflict of interest.

## Publisher’s Note

All claims expressed in this article are solely those of the authors and do not necessarily represent those of their affiliated organizations, or those of the publisher, the editors and the reviewers. Any product that may be evaluated in this article, or claim that may be made by its manufacturer, is not guaranteed or endorsed by the publisher.

## Supplementary Material

Supplementary Figure 1 Comparison of Multilocus Sequence analysis (MLSA) Tree, Average Nucleotide Identity (ANI) Tree, and Parsnp Tree. MLSA, ANI, and Parsnp trees, containing 39 *X. perforans* strains, were developed to compare and identify known groups and clades. **(A)** Concatenation of five house-keeping genes, *lepA, gyrB, lacF, gapA*, and *gltA*, was used to build the MLSA phylogenetic tree. **(B)** Whole genome sequence was used for the ANI phylogenomic tree. **(C)** Whole Genome sequence was used for Parsnp phylogenomic tree. Strains are denoted by the name, location of isolation, year, race-type, and known mutation in *avrXv3*.
