# Supplementary figures and images for: Migration Drives the Replacement of Xanthomonas perforans Races in the Absence of Widely Deployed Resistance
Source: Front Microbiol. 2022 Mar 18;13:826386. doi: 10.3389/fmicb.2022.826386 (PMC8971904; doi:10.3389/fmicb.2022.826386)

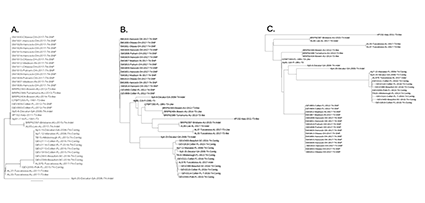

Supplement: Supplementary Figure 1 — Comparison of Multilocus Sequence analysis (MLSA) Tree, Average Nucleotide Identity (ANI) Tree, and Parsnp Tree. MLSA, ANI, and Parsnp trees, containing 39 X. perforans strains, were developed to compare and identify known groups and clades. (A) Concatenation of five house-keeping genes, lepA, gyrB, lacF, gapA, and gltA, was used to build the MLSA phylogenetic tree. (B) Whole genome sequence was used for the ANI phylogenomic tree. (C) Whole Genome sequence was used for Parsnp phylogenomic tree. Strains are denoted by the name, location of isolation, year, race-type, and known mutation in avrXv3. [file Image_1.TIF]
